# Apical debris extrusion during instrumentation of oval root canals in primary teeth using manual versus motorized files: an ex vivo study

**DOI:** 10.1038/s41598-021-83522-4

**Published:** 2021-02-16

**Authors:** Bhaggyashri A. Pawar, Ajinkya M. Pawar, Jatin Atram, Alexander Maniangat Luke, Anuj Bhardwaj, Anda Kfir, Zvi Metzger, Dian Agustin Wahjuningrum

**Affiliations:** 1 Department of Oral Health and Advanced Dentistry, Sir H. N. Reliance Foundation Hospital and Research Centre, Mumbai, Maharashtra India; 2 Department of Conservative Dentistry and Endodontics, Nair Hospital Dental College, Mumbai, Maharashtra India; 3 Department of Surgical Sciences, College of Dentistry, Ajman University, Ajman, UAE; 4 Department of Conservative Dentistry and Endodontics, College of Dental Sciences and Hospital, Rau, Indore, India; 5 Department of Endodontology, The Goldschleger School of Dental Medicine, Tel Aviv University, Tel Aviv, Israel; 6 Department of Conservative Dentistry, Faculty of Dental Medicine, Universitas Airlangga, Jl. Moestopo 47, Surabaya, Jawa Timur Indonesia

**Keywords:** Paediatric research, Preclinical research

## Abstract

This study aimed to assess the apical extrusion of debris during instrumentation of primary canines using three endodontic file types. Forty-five extracted primary canines were randomly assigned to three instrumentation groups (*n* = 15): Hand K-files; and the motorized Kedo-S files and XP-endo Shaper files. The apically extruded debris produced during the procedure was collected and dried in pre-weighed Eppendorf tubes, and the mass of debris was calculated. The time required for the endodontic procedure was also recorded. Analysis of variance (ANOVA) and Tukey’s post hoc test were used with a significance level set at 5%. XP-endo Shaper and Kedo-S files extruded significantly less debris compared with hand K-files with means of 0.84 ± 0.31 and 1.20 ± 0.67 mg respectively, compared to 2.13 ± 0.31 mg (*p* < 0.0001). No significant difference was found between the two motorized files. Less time was required to complete the procedure with the XP-endo Shaper compared to the hand K-files (*p* < 0.0001) and Kedo-S files (*p* < 0.0001). Within the limitations of the present study, it may be concluded that motorized files extruded less debris and required less instrumentation time compared to traditional K-files, which could benefit paediatric patients with root canal treatment needs.

## Introduction

Pulpectomy and root canal procedures remain the first treatment option in primary teeth with pulpal involvement^1^. This therapy aims to heal and/or maintain the involved periapical tissue and salvage the teeth until the eruption of permanent successors^2^.

Traditionally, root canal shaping was achieved with hand instruments, such as K-files. However, the use of such files may result in canal aberrations, perforations, inadequate cleaning and transportation of the root canal. Hand instrumentation also requires a rather long chair time for patients^3^.

Kedo-S paediatric nickel titanium (NiTi) rotary files (Reeganz Dental Care, Chennai, India) were introduced to overcome some of the above problems^4^. The files are shorter than the common NiTi rotary files (total length 16 mm), and their flexibility allows better adaptation to curvatures that are often found in primary teeth^4^. The file has a triangular cross-section and a non-cutting tip with a 12-mm long active part and a taper that gradually changes from 0.04 to 0.08. The Kedo-S file is used as a single file system.

Root canals with an oval cross-section are common in primary dentition^5^. Oval canals present a challenge to all rotating files that have a central metal core. Rotating endodontic files have a tendency to create a space with their own shape with a round cross-section. Such root canal preparation may leave uninstrumented recesses in which tissue remnants and debris may be left untouched^6^. Recently introduced XP-endo Shaper (FKG Dentaire, La Chaux-de-Fonds, Switzerland) was specifically designed to meet the challenge of oval root canals^7^. The file has a size 30 “booster tip” design that includes a combination of a smooth bullet-shaped tip followed by six cutting edges and a smooth transition from the tip base to the helical shaft made of a size 30 wire with a 0.01 taper. This file is made from a special thermo-mechanically treated shape-memory Ni–Ti alloy and has a snake-like shape at room temperature (martensite phase). When exposed to 37 °C, a transfer to the austenite phase occurs, and the snake-like shape is enhanced and assumes a greater rotational envelope of motion that is equivalent to size 30 tip with a 0.04 taper. This dimension is achieved with no solid central part^8^. When used at 800 rounds per minute and with long in-and-out pecking motions, the tip of the file enters repeatedly into and cleans the recesses of the oval canal. The XP-endo Shaper is a single file system.

All endodontic instrumentation methods have a tendency to push debris through the apical foramen and into the periapical tissues^9–11^. Such debris may consist of necrotic pulp tissue, dentin chips and bacteria. Extrusion of such debris may induce postoperative pain and inflammation and may inhibit periapical healing^9–11^. A growing body of literature has recognized the importance of reducing apically extruded debris^9–11^. Estimating the root canal length just short of the apex, using an electronic apex locator, would also seem helpful in reducing the chance of extruding debris beyond the apex. Using such a device will not be influenced by tooth type, root canal type, status of the periapex, or clinical condition^12^.

The extent of debris extrusion and the time required for instrumentation of the canals of primary teeth using adaptive XP-endo Shaper files have not been reported to date. Very few studies have been conducted on debris extrusion by Kedo-S paediatric rotary files^13,14^.

The present study aimed to measure and compare the amount of apically extruded debris using Kedo-S paediatric rotary and the new XP-endo Shaper files, both of which are operated by an endo-motor (motorized) and to compare both to traditional hand-operated K-files. Measuring the time required to complete the bio-mechanical preparation by these three files was a second goal of the present study.

This study examined the following two-fold null hypothesis: (a) there is no difference in apically extruded debris between hand-operated K-files and the two motorized systems and (b) the time required for completing the procedures is not different among the three tested file systems.

## Materials and methods

### Sample allocation and ethical approval

This ex vivo study was approved by the College of Dental Science & Hospital Ethics Committee (Certificate CDSH/IEC/2018-2019/004). Forty-five primary canines were selected from a pool of recently extracted primary teeth. The roots of the teeth were cleaned using periodontal curettes, and the teeth were stored in water with 5% thymol at 4 °C until use in the experiments^15^. This sample size was calculated by projecting the power, effect size, and significance level as 0.91, 0.697, and 0.05, respectively, based on the results of a previous study^13^.

### Inclusion criteria

The inclusion criteria were a single root canal and foramen as confirmed by bucco-lingual and mesio-distal radiographs. These criteria also included no evidence of resorption, a closed apex, and a long through short canal diameter ratio of ≥ 2 at 5 mm from the apex, as measured from bucco-lingual and mesio-distal radiographs^16^. Access cavities were made, and canal patency was assessed for all samples by inserting a #10 K-file (Mani, Tokyo, Japan) until visible at the apical foramen. Since none of the teeth presented with apical resorption the working length (WL) was established as 1 mm short of the apex^17–19^. The clinical crowns of the teeth were further ground using a high-speed diamond straight fissure bur under air–water spray to obtain a total length of 15 mm and WL 14 mm in the standardization of all samples. The 45 samples were then sequentially numbered and randomly divided (www.random.org) into 3 groups (*n* = *15)* for cleaning and shaping by one of three methods, including hand K-files, Kedo-S paediatric rotary files, and XP-endo Shaper files.

### Experimental model

**Figure 1:**
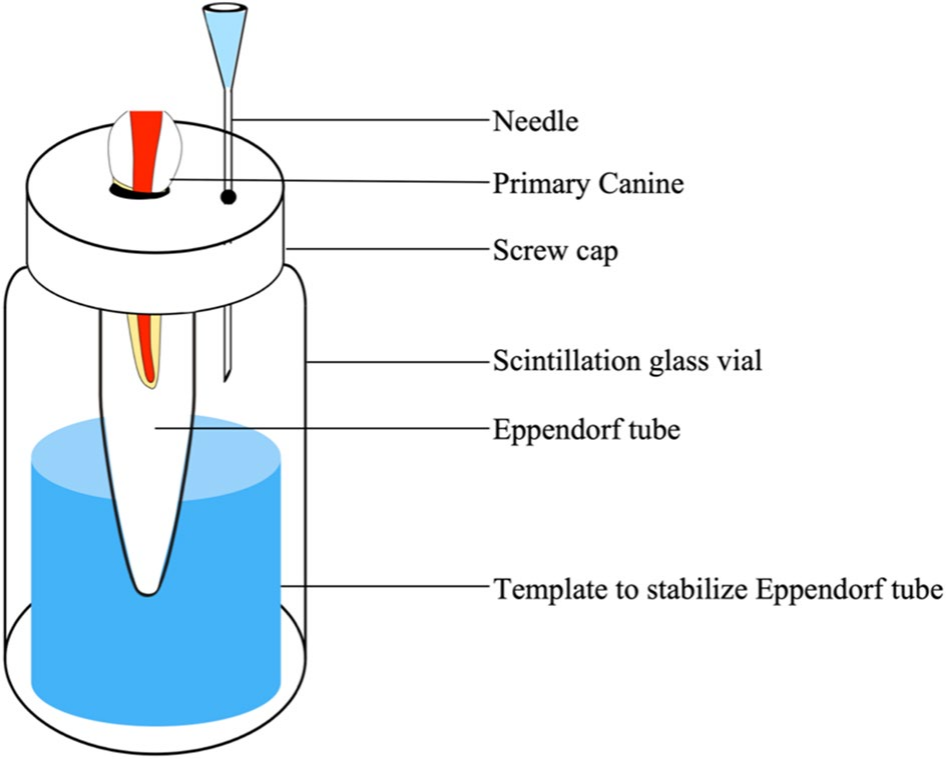
Schematic presentation of the apparatus used to obtain apically extruded debris.

The model was proposed by Myers and Montgomery^16^, with modifications to the apparatus which were suggested by Kfir et al.^11^ were used to measure the apical extrusion of debris (Fig. 1). Forty-five 1.5-ml Eppendorf tubes (IndoSurgical, New Delhi, India) were obtained, and the caps of the tubes were separated. The tubes without caps were weighed to 10^−5^ g precision using a microbalance (Sartorius, Hamburg, Germany). Additionally, three consecutive weight measurements were acquired per tube, and the mean value was recorded. Subsequently, fifteen tubes were assigned to each of the three groups.

Forty-five glass scintillation vials (Cole-Parmar, Mumbai, India) were acquired, and holes were created in the caps where a primary canine was inserted, with the apex facing down, to the level of the cementoenamel junction. The teeth were secured in place with a flowable composite (Filtek Supreme; 3 M ESPE, St Paul, MN, USA). Additionally, a 25-gauge needle (BD India, Gurgaon, India) was also placed and secured in the cap to equalize air pressure in and out of the vial. A small holding template was created on the bottom of the vial using silicon impression material (Coltène/Whaledent, Langenau, Germany) to hold and stabilize each Eppendorf tube so that when the caps were fitted onto the vials, the root tip was located within the Eppendorf tube without touching its walls. The glass vials were then covered with a rubber dam (CricDental, Mumbai, India) such that the operator was blocked from viewing the debris extrusion and tooth during root canal preparation. (Fig. 1) The entire apparatus was exclusively handled by the scintillation vial.

### Root canal instrumentation Group 1: hand K-files

The root canals were instrumented to 14 mm from the coronal reference point using quarter turn and pull motion. Stainless steel hand operated K-files were utilized in a sequence of #15/0.02, #20/0.02, #25/0.02, and #30/0.02 (Mani, Tokyo, Japan). Irrigation was performed before and after each file using a syringe and needle (NaviTip 31G; Ultradent, South Jordan, UT, USA). The needle was inserted at each stage and withdrawn 2 mm short of where it engaged at this stage or 2 mm short of WL. One ml of distilled water was used for irrigation at each stage with a total irrigation volume of 4 ml per tooth.

### Root canal instrumentation Group 2: Kedo-S paediatric rotary files

Kedo-S is a single file system, and the E1 file used in the present study has a #30 tip and gradually changing taper from 0.04 to 0.08 (Reeganz Dental Care, Chennai, India). The 16-mm long files were used in rotary motion of 250 rpm and 2 Ncm torque, powered by an electronic endomotor (X-Smart Plus; Dentsply Maillefer, Ballaigues, Switzerland) according to the manufacturer’s instructions. No pre-flaring was required. Gentle in-and-out motions were used to reach WL. Once the file met resistance, the file was retracted, cleaned with a gauze and applied again. Once the file reached WL, apical patency was verified, and the file used with five in-and-out motions to WL, as per manufacturer’s instructions. Irrigation was performed using a syringe and needle (NaviTip). The needle was inserted at each stage and withdrawn 2 mm short of where the needle engaged at this stage or 2 mm short of WL. Irrigation was done with distilled water which was applied at four stages of the procedure: 1 ml of distilled water was used for irrigation before insertion of the file into the canal, 1 ml after the first withdrawal of the file (for cleaning), 1 ml after reaching WL (before the final 5 in-and-out movements) and 1 ml after completing the instrumentation with a total irrigation volume of 4 ml per tooth.

### Root canal instrumentation Group 3: XP-endo Shaper

The root canals were cleaned and shaped using a 21 mm XP-endo Shaper file (FKG Dentaire, La Chaux-de-Fonds, Switzerland), as a single file, following manufacturer’s instructions: The file was operated at 800 rpm and 1 Ncm torque, powered by an endomotor (X-Smart Plus), until WL was reached. Initially, the file was placed passively into the canal until resistance was encountered, then the tip retracted 2 mm, and the endomotor activated. The file was then used 4–5 times by the application of long gentle strokes towards WL. Once the file reached WL the file was withdrawn and cleansed, the apical patency verified, the canal flooded with warm (37 °C) distilled water, and then the file reused for an additional 15 in-and-out strokes to WL, as recommended by the manufacturer. Irrigation was performed using a syringe and needle (NaviTip). The needle was inserted at each stage and withdrawn 2 mm short of where it engaged at this stage or 2 mm short of WL. The irrigant was warmed to and kept at 37 °C (using a temperature controlled water bath), to allow transition of the files from the martensite to the austenite phase. Irrigation was done at four stages during the procedure: 1 ml of distilled water was used for irrigation before insertion of the file into the canal, 1 ml after the first 5 strokes, 1 ml after reaching WL (before the final 15 in-and-out movements) and 1 ml after completing the instrumentation, with a total irrigant volume of 4 ml per tooth.

Irrigation in all three groups was done at a flow rate of about 0.3 ml per minute.

A new file was used to prepare each canal, and a single operator performed all the experiments to avoid inter-operator variability. The operator was an experienced paediatric dentist who had intensive experience with the use of each of the three endodontic file systems.

### Assessment of apically extruded debris

Following root canal preparation, the caps of the vials were unscrewed and the Eppendorf tubes removed. The surface of the root was washed with 1 ml of distilled water to collect adhered debris into the Eppendorf tubes. Then, the tubes were placed in an incubator at 70 °C for 5 days to permit the evaporation of all moisture. The weight of each tube was determined as the mean weight from three consecutive weights in milligrams. The weight of the tube before the procedure was subtracted from the above, thus resulting in the weight of extruded debris ^9,11^.

### Assessment of time required for instrumentation

The duration of the procedure was recorded by the operator performing the study using a digital stopwatch. The starting point was the first insertion of the file into the canal, and the end point was the end of the final irrigation with distilled water.

### Statistical analysis

The amount of extruded debris, alongside the time required for the instrumentation, were analysed statistically by the implementation of a one-way analysis of variance (ANOVA) followed by Tukey’s post hoc test for the execution of multiple comparisons. The level of significance was set at 5%, and all analyses were performed with Statistical Package for the Social Sciences version 20 for Mac (SPSS, IBM, Chicago, IL, USA).

## Results

### Debris extrusion

The amount of apically extruded debris by each of the three file systems is presented in Table 1. The mean weights (± SD) were 2.13 (± 0.46) mg in Group 1 (Hand K-files), 1.2 (± 0.67) mg in Group 2 (Kedo-S) and 0.84 (± 0.31) mg in Group 3 (XP-endo Shaper). Significant differences among the groups were identified (ANOVA, *p* < 0.0001). Tukey’s post hoc test revealed that the amount of extruded debris in both the XP-endo Shaper and Kedo-S groups was significantly less than the amount of debris extruded in the Hand K-file group (*p* < 0.0001). However, the amount of debris in the two motorized file groups did not differ significantly from each other (Table 1).

**Table 1:** Analysis of variations in the mean weight of apically extruded debris using three different file systems.

Group	Files	Sample size	Mean weight of extruded debris in milligrams (± SD)	Tukey HSD *p* values
1	Hand K-files	15	2.13 (± 0.46)	0.000*; 0.000**
2	Kedo-S	15	1.20 (± 0.67)	0.127***
3	XP-endo shaper	15	0.84 (± 0.31)	

*Comparison between Hand K-files and Kedo-S, **Comparison between Hand K-files and XP-endo Shaper, ***Comparison between Kedo-S and XP-endo Shaper.

*P* value of less than 0.05 was considered as statistically significant.

### Time required for instrumentation

The mean time required to complete the procedures was 7.33 ± 1.2 min in Group 1, 4.61 ± 0.73 min in Group 2, and 2.38 ± 0.58 min in Group 3 (Table 2). A significant difference was found among the groups (ANOVA, *p* < 0.001), and Tukey’s post hoc* test* indicated that significantly less instrumentation time was required in the XP-endo Shaper group compared to the other two groups (*p* < 0.001).

**Table 2:** Analysis of variations in the time required to complete the procedure using three different file systems.

Group	Files	Sample size	Mean time in minutes (± SD)	Tukey HSD *p* values
1	Hand K-files	15	7.33 (± 1.20)	0.000*; 0.000**
2	Kedo-S	15	4.61 (± 0.73)	0.000***
3	XP-endo shaper	15	2.38 (± 0.58)	

*Comparison between Hand K-files and Kedo-S, **Comparison between Hand K-files and XP-endo Shaper, ***Comparison between Kedo-S and XP-endo Shaper.

*P* value of less than 0.05 was considered as statistically significant.

## Discussion

Data on debris extrusion during the shaping of root canals of primary teeth with the new adaptive XP-endo Shaper were previously non-existent to the best of our knowledge. The present results indicate that both XP-endo Shaper and Kedo-S procedures were associated with less apically extruded debris than the use of hand K-files (*p* < 0.0001). Therefore, the first null hypothesis was rejected.

The findings broadly support previously reported studies^1–3,9,13,14,20,21^. Apical extrusion of debris is caused by accumulation of debris in the apical part of the canal where it may be pushed beyond the apex^22^. The high amount of debris extruded by the K-files could result from the filing motion, which may act as a piston when the files are engaged in the apical third of the canal^23^. Furthermore, the K-files have a constant (0.02) taper, which may provide less space in the apical part for dentin chips and debris that have to be transported coronally; consequently, the debris may be pumped apically^24^.

**Figure 2:**
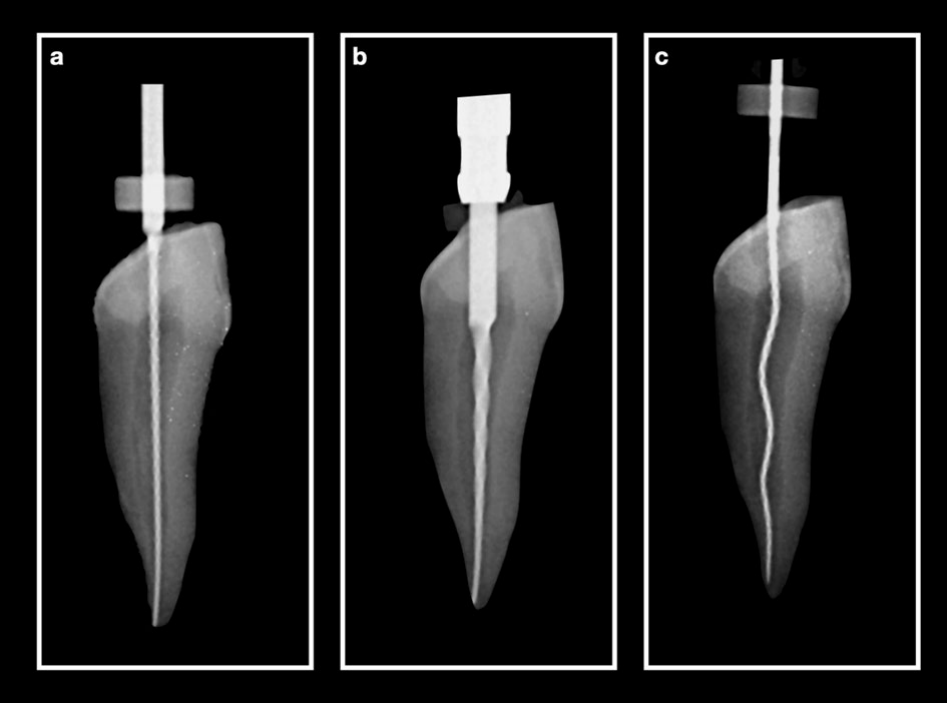
Radiographic image of the three files in a primary canine (mesio-distal projections) (**a**) #30 hand K-file, (**b**) Kedo-S E1 rotary file, and (**c**) XP-endo Shaper.

It could be expected that the XP-endo Shaper will cause less debris extrusion than the rotary Kedo-S files due to the differences in their shape and mode of action. The Kedo-S rotary file with its bulky core (size 30 and 0.04 to 0.08 taper, Fig. 2) fills the apical part of the canal and leaves little space for the suspension of debris compared to the loose space around the XP-endo Shaper (Size 30 and 0.01 taper, Fig. 2). Furthermore, when rotating at 800 rpm and at 37 °C, the file has an envelope of motion with a 0.04 taper, the centre of which is hollow in contrast to the solid metal core in the Kedo-S file.

The method of debris removal by the XP-endo Shaper and the Kedo-S files is also completely different. The XP-endo Shaper suspends the debris and carries it coronally with a tornado-like movement of the irrigant created by the speed of rotation (800 rpm) and the snake-like shape of the file^7,22^. Such suspension and transportation of debris was expected to be more efficient than forcing debris coronally by the rotation of the flutes of the Kedo-S file^7,22^. Despite these differences in shape and mode of action and even though the mean amount of apically extruded debris was apparently reduced with the XP-endo Shaper, the difference in apical extrusion of debris between the groups was not statistically significant (Table 1). The reason could be that the piston effect at the apical 2–3 mm of the pecking in-and-out motion used with both instruments had more influence than the potential benefit of the way the XP-endo Shaper file is transporting debris coronally.

The XP-endo Shaper was selected for the present study as it is a new device specially designed to overcome a major specific problem in root canal instrumentation. Many roots in the primary dentition have root canals with an oval cross-section^5^. Most current motorized files, including the Kedo-S file, have a solid metal core and tend to create a space with a circular shape in every root canal, which may limit the cleaning ability of the endodontic procedure. Esentürk et al.^25^ recently demonstrated that when rotary files are used in primary teeth, 60% of the canal wall area remains un-instrumented. Canal preparations with a round cross-section are likely to leave tissue, bacteria and debris in the un-instrumented recesses of oval canals, thus jeopardizing treatment prognosis^26^.

A dominant benefit of using motorized files in primary teeth is reduction of the time required to complete the endodontic procedure^21,27^. Reducing the time may be especially beneficial when children are treated, as it may enhance patient cooperation. The present results indicate that the use of XP-endo Shaper required 68% less time than hand instrumentation with K files (*p* < 0.0001). The Kedo-S file also reduced instrumentation time by 37%, but the procedure required more time than that with the XP-endo Shaper (*p* < 0.0001). Thus, the second null hypothesis had also been rejected.

The difference in time required between the two motorized procedures may have resulted from the mode by which each of the files was used to reach WL. The Kedo-S file had to remove a large amount of dentin with its rather bulky active part before its non-cutting tip may reach the working length. Consequently, one has to stop at least once or twice to remove the accumulated debris from the file’s flutes if one does not want to apply excessive force during this procedure. The tip of the XP-endo Shaper has a unique design that makes reaching WL very fast with almost no pressure. The tip is divided into two parts. The apical part of the tip has a non-cutting bullet shape, which then changes into 6 cutting blades that then merge into the thin shaft with a 0.01 taper. It seems that these features allow the XP-endo Shaper file to reach WL easily and quickly with minimal resistance. The XP-endo Shaper file is not expected to shape the canal but rather clean it with its tip entering the canal irregularities with each of the following 15 long pecking strokes that are recommended by the manufacturer. Further studies with micro CT may be required to examine the effectiveness of cleaning the canals of primary teeth by these two devices.

It must be kept in mind that the present study was conducted using only single rooted primary canines with straight roots and no apical resorption. The effect of apical resorption, which is common in primary teeth^28^, on apical extrusion of debris should be addressed in future studies. Naturally, the time required to complete treatment of three rooted primary molars may be longer than that of a single rooted canine. Primary molars often have curved root canals with oval cross-sections^5^; thus, further studies on the use of XP-endo-Shaper in primary molars may be required using both micro CT and debris extrusion measurements while also measuring the time required to complete the procedure.

## Conclusions

Within the limitations of the present study, it may be concluded that motorized files extruded less debris and required less instrumentation time compared to traditional K-files, which could benefit paediatric patients with root canal treatment needs.
